# Multifunctional Dyeing Process and Characterization of Silk Fabric Based on Natural Colorant of Rhubarb

**DOI:** 10.3390/molecules31071165

**Published:** 2026-03-31

**Authors:** Xuzhi Sun, Ge Pan, Xiaojuan Li, Qingru Huang, Xiang Ma, Mingfei Sheng, Maoli Yin

**Affiliations:** 1 School of Textile and Garment, Anhui Polytechnic University, Wuhu 241000, China; 2 School of Textile Garment and Design, Suzhou University of Technology, Suzhou 215500, China; 3 Key Laboratory of Clothing Materials of Universities in Fujian, Quanzhou Normal University, Quanzhou 362000, China

**Keywords:** rhubarb, silk, dyeing, UV resistance, antibacterial properties

## Abstract

To promote the application of natural dyes in eco-textiles and develop multifunctional silk fabrics, this study optimized the extraction of functional pigments from rhubarb and investigated their dyeing performance and functional properties on silk. The optimal extraction conditions were determined as pH 11, 80 °C, 50 min, with three extraction stages. The optimized direct dyeing parameters for silk fabrics were: dye bath pH value of 7, bath ratio of 1:40, dye solution concentration of 5%, and dyeing at 80 °C for 60 min. Post-dyeing metal ion mordanting significantly regulated the hue and dyeing depth of fabrics, with ferrous sulfate mordanting demonstrating the most ideal effect, enabling fabrics to exhibit deep gray coloration and a substantial increase in K/S value. The dyed silk exhibited significantly enhanced Ultraviolet (UV) protection (UPF 18.72 for direct dyeing, reaching 29.80 after Fe^2+^ mordanting) and antibacterial activity (inhibition rates of 69.26% and 77.49% against *Escherichia coli* (*E. coli*) and Staphylococcus aureus (*S. aureus*), respectively, exceeding 95% after Fe^2+^ treatment). This work demonstrates that rhubarb dyeing can produce functional silk with excellent UV-blocking and antibacterial properties, supporting its potential in ecological textiles.

## 1. Introduction

In recent years, the growing emphasis on low-carbon environmental protection and increasing consumer health awareness have heightened the demand for eco-friendly dyeing and finishing technologies in the textile industry [1,2]. Although synthetic dyes offer a wide broad color spectrum and high dyeing efficiency, they are often associated with toxicity, poor biodegradability, and environmental pollution during both production and application, posing serious threats to the environment and human health [3,4,5]. In contrast, natural dyes derived from plant, animal, or mineral sources, present a promising alternative due to their non-toxicity, biodegradability, and favorable environmental compatibility. Moreover, many natural dyes possess functional properties such as UV protection, antibacterial activity, and antioxidant effects [6,7,8,9], which has revived significant interest in their application across both research and industry. Accordingly, numerous studies have focused on dyeing and functionalizing textiles using natural materials from various sources to develop high value-added textiles [10,11,12].

Silk fabric, often celebrated as the “second skin” of humans, is a natural fiber prized for its lightness, softness, and fineness. Its smooth luster, moisture absorption, breathability, and excellent biocompatibility make it highly suitable for high-end apparel and medical applications [9,13,14,15]. Despite the maturity of industrial processing techniques, challenges remain in silk dyeing. Conventional dyeing typically requires high temperatures (around 95 °C) to promote dye adsorption and diffusion, which can lead to fiber strength loss and surface abrasion [16]. Moreover, silk is commonly colored with acid dyes that require fixing agents, and it inherently lacks sufficient UV resistance and antibacterial activity properties often imparted using synthetic auxiliaries [17]. These chemical dyes and additives compromise the appeal of silk and raise environmental and health concerns [4]. Therefore, applying functional natural dyes to silk to simultaneously impart color and functionality holds considerable practical value.

To date, numerous studies have explored the use of natural extracts for dyeing and functionalizing silk. These include plant-based colorants from leaves, roots, and fruits, such as indigoids, carotenoids, quinones, flavonoids, and alkaloids, as well as animal-derived dyes like cochineal and lac [18,19,20]. Rhubarb, a traditional Chinese medicinal herb, contains a variety of functional anthraquinone derivatives in its extract, including emodin, chrysophanol, rhein, and aloe-emodin [21,22,23]. Based on their molecular structures, these bioactive compounds are theoretically expected to exhibit broad-spectrum antibacterial effects and UV-blocking capability based on their molecular structure [24,25,26]. Applying rhubarb functional pigments to silk dyeing not only introduces distinctive coloration but also adds valuable health-promoting functions. Thus, utilizing rhubarb extract as a dye to develop non-toxic, low-pollution dyeing processes aligns with the goals of ecological sustainability, environmental protection, and healthy development.

**Figure 1:**
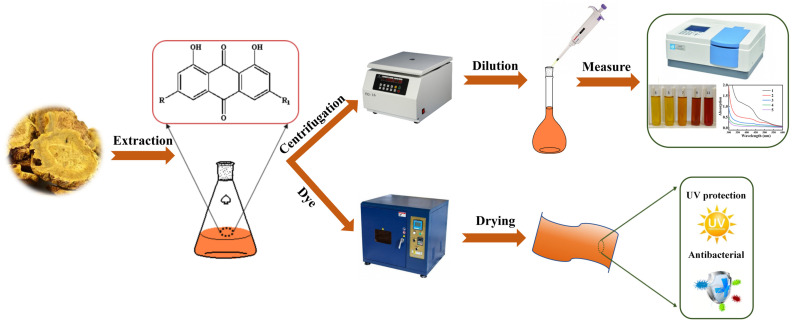
Schematic presentation of rhubarb pigment extraction and dyeing process.

This study focuses on rhubarb as a natural dye source. We optimized its pigment extraction process and systematically investigated the effects of dyeing parameters and post-mordanting with metal ions on the colorimetric properties, UV resistance, and antibacterial performance of dyed silk (Figure 1). The aim is to provide a theoretical foundation and technical support for developing high-performance ecological silk textiles while expanding the application scope of natural dyes.

## 2. Results and Discussion

### 2.1. Optimization of Extraction Process for Rhubarb Functional Pigment

**Figure 2:**
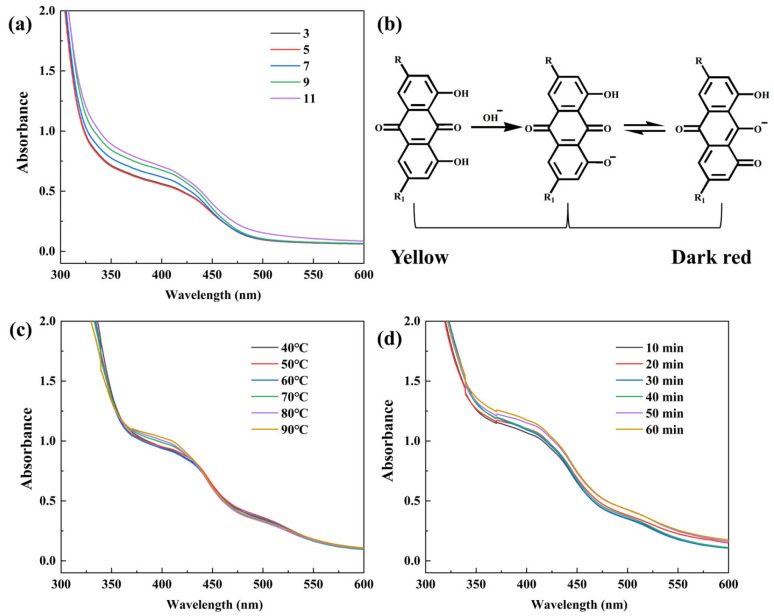
Analysis of the pigment extraction process of rhubarb pigment: (**a**) pH value; (**b**) ionization of rhubarb pigment at different pH; (**c**) temperature; (**d**) time.

The pH of the extraction solvent significantly influenced the solubility and color development of rhubarb pigment. With extraction temperature fixed at 80 °C and time at 60 min, the absorbance in the visible region gradually increased with rising pH, and the color deepened from yellow under acidic conditions to reddish-brown under alkaline conditions (Figure 2a). At pH 3, absorbance was lowest and essentially overlapped with the curve at pH 5. The spectra at pH 7 and pH 9 were relatively close, while the maximum absorbance was achieved at pH 11. This is attributed to the ionization of hydroxyl and carboxyl groups in the pigment molecules under alkaline conditions, forming anionic structures [17,27] (Figure 2b), which enhances solubility and electron-donating ability, thereby promoting electron transition and color deepening. Although pH 11 may not be the absolute optimum from an extraction yield standpoint, it was selected for subsequent extractions considering practical efficiency.

Figure 2c shows the influence of extraction temperature at pH 11 and 50 min. As temperature rose from 30 °C to 90 °C, absorbance at 400 nm increased continuously. Higher temperatures promote molecular movement and dissolution–diffusion, but balancing energy consumption and extraction efficiency, 80 °C was selected as the suitable temperature.

The effect of extraction time under pH 11 and 80 °C is shown in Figure 2d. Within 10–60 min, absorbance increased gradually and leveled off after 50 min, indicating that extraction approached equilibrium. Considering energy savings and efficiency, 50 min was chosen as the optimal extraction time.

**Figure 3:**
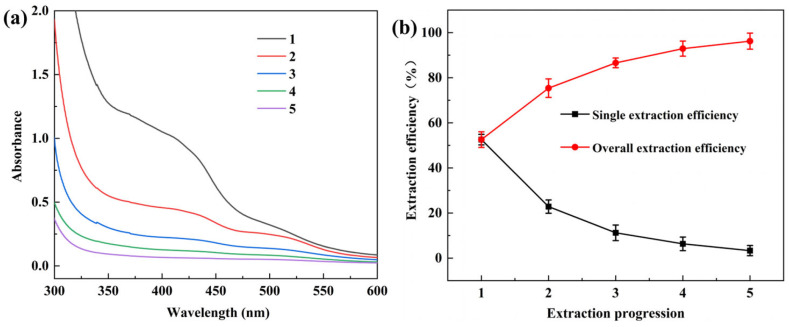
(**a**) Effect of rhubarb extraction count on absorbance; (**b**) single extraction efficiency and overall extraction efficiency of different extraction stages.

Using the optimized conditions (pH 11, 80 °C, 50 min), the effect of extraction stages was examined. Figure 3a and Figure 3b respectively demonstrate the single extraction efficiency and overall extraction efficiency across different extraction stages under varying extraction cycles. After three stages, absorbance decreased to 0.2241, with a total extraction yield of 86.57%. At the fourth stage, the extract color faded noticeably, with a single-stage yield of only 6.35%. After the fifth stage, the extract was nearly colorless (absorbance 0.0665, yield 3.32%). Based on energy savings, solvent economy, and extraction efficiency, three extraction stages were determined as optimal.

In summary, the optimal extraction process for rhubarb functional pigment is: pH 11, temperature 80 °C, time 50 min, and three extraction stages.

### 2.2. Optimization of Dyeing Process for Silk Fabric

**Figure 4:**
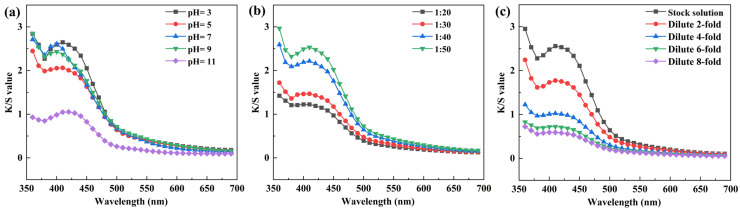
Effect of (**a**) pH, (**b**) liquor ratios and (**c**) dilution ratio on the K/S value of silk fabrics.

Data analysis indicated that dye bath pH significantly affected the apparent color depth (K/S) of dyed silk. As shown in Figure 4a, the highest K/S value (2.6474) was obtained at pH 3. This is mainly because, when the pH is below the isoelectric point of silk (3.5–5.2), amino groups on the fiber surface adsorb H^+^ and become positively charged, enabling ionic bonding with anionic groups in the rhubarb dye [15,20]. Moreover, under acidic conditions, pigment aggregation is higher and hydroxyl-group dissociation is weaker, which also favors dye uptake [28]. At pH 5, the K/S value decreased. At this pH, close to the lower limit of the fiber’s isoelectric point, the positive charge on the fiber surface is reduced, weakening ionic interactions. Additionally, dissociation of carboxyl and hydroxyl groups in the pigment is suppressed, lowering solubility and hindering dyeing. At pH 7, the maximum absorption wavelength shifted to 400 nm, and the K/S value recovered notably compared with pH 5, approaching that at pH 3. This suggests that under near-neutral conditions, gradual dissociation of carboxyl and hydroxyl groups improves solubility, while electrostatic repulsion between dye and fiber remains insignificant, resulting in better dye uptake. When pH was further increased to 9 and 11, K/S values continued to decline, with a pronounced drop at pH 11, and the maximum absorption wavelength red-shifted to 420 nm. Under strongly alkaline conditions, the high degree of pigment ionization creates strong electrostatic repulsion with the negatively charged fiber surface, severely hindering dye adsorption and leading to reduced color depth and chroma [27]. Therefore, pH 7 was selected as the optimal parameter, offering good dye solubility and moderate charge interaction with the fiber.

As shown in Figure 4b, the K/S value of dyed fabric gradually increased with higher liquor ratios, and the maximum absorption wavelength remained stable at 410 nm for all samples. This trend is attributed to better contact between dye liquor and fabric at higher liquor ratios, which promotes diffusion and adsorption of rhubarb pigment into the fiber, thereby improving dye uptake [29,30]. Further analysis of the spectral curves shows that when the liquor ratio increased from 1:20 to 1:30, the rise in K/S value was small. However, increasing the liquor ratio from 1:30 to 1:40 caused a significant jump of 0.7481 in K/S, indicating that the liquor ratio had a notable influence on dyeing effectiveness within this range. When the liquor ratio was further raised to 1:50, the increase in K/S slowed, suggesting diminishing returns on color yield. Considering dyeing performance, dye utilization efficiency, and process economy, a liquor ratio of 1:40 was selected for subsequent optimization, as it ensures satisfactory K/S and color characteristics while saving dye chemicals and reducing processing load.

The effect of dye concentration (expressed as dilution ratio) on the dyeing outcome was investigated using the apparent color depth (K/S) as the main indicator; the corresponding absorption spectra are shown in Figure 4c. As the dilution ratio increased (i.e., concentration decreased), the K/S value of dyed fabric gradually declined. This is primarily because the effective concentration of rhubarb pigment in the dye bath decreases, reducing the total amount of pigment available for fiber uptake, leading to lighter shades that approach the original white of silk. Further analysis of spectral curves and K/S changes reveals that within the 0–4× dilution range, the drop in K/S was most significant, indicating that concentration variation greatly affects dyeing depth in this stage. Beyond 4× dilution, the decline in K/S gradually leveled off, and the fabric appearance turned noticeably pale, suggesting that dye uptake had reached a low level. In this experiment, the stock solution concentration was 5% (material-to-liquid ratio 1:20). To achieve deeper dyeing depth and fuller hue, the undiluted stock solution was selected as the optimal dye concentration.

In summary, the optimal dyeing process for rhubarb functional pigment is: pH 7, bath ratio 1:40, 5% dye concentration.

### 2.3. Post-Mordanting with Metal Ions

**Figure 5:**
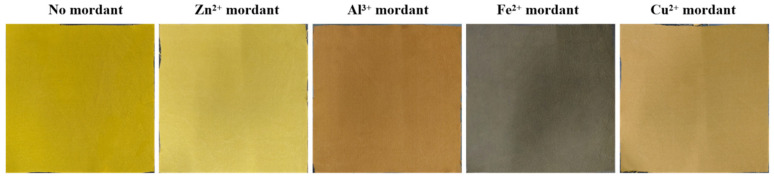
Representative images of silk fabrics.

**Figure 6:**
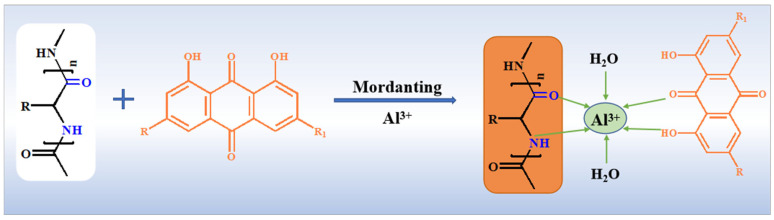
Formation and fixation mechanism of dyeing silk fabrics with rhubarb extract using mordant.

Post-mordanting with four metal ions (Zn^2+^, Al^3+^, Cu^2+^, Fe^2+^) was performed on rhubarb-dyed silk. Color changes were characterized using absorption spectra and CIEL*a*b*/CIEL*c* values, as shown in Figure 4 and Table 1. Compared with direct dyeing, post-mordanting generally deepened the shade while altering color depth. Al^3+^ and Zn^2+^ mordants had a modest influence on fabric hue, which remained similar to that of direct dyeing. Cu^2+^, and Fe^2+^ mordants significantly increased the K/S value. After Fe^2+^ treatment, the mordanted fabric appeared darker and grayer; the shortened distance of the chromaticity coordinates (a, b) from the origin also confirms the darker shade (Figure 5). Rhubarb dye belongs to the anthraquinone class; the hydroxyl groups at the α-position of its anthraquinone structure are reactive and can form stable chelate rings with carbonyl groups [30,31]. Interaction among metal salts, rhubarb dye, and silk leads to the formation of metal–dye complexes, as illustrated in Figure 6. The color change arises from the unfilled d-orbitals in transition metals acting as ligands in the metal–dye complex.

### 2.4. Performance Analysis of Dyed Silk Fabric

#### 2.4.1. Color Fastness of Dyed Fabrics

During use, silk fabrics are subjected to various mechanical actions such as rubbing, washing, and light exposure. In practical applications, silk fabrics have different color fastness requirements depending on their intended use. Dyeing fabrics with natural dyes often results in poor color fastness of the dyed textiles, and a common approach to overcoming this issue is the use of mordants. In this study, silk fabrics were dyed with 5% rhubarb extract and mordanted with 3% owf. mordant. The color fastness of the resulting dyed silk fabrics is shown in Table 2. As can be seen from Table 2, mordant dyeing significantly improved the color fastness of silk. The washing fastness and rubbing fastness of the fabrics were both enhanced, which can be attributed to the formation of complexes between the mordant, silk fibers, and rhubarb pigments during the mordanting process, thereby increasing the binding affinity between the dye and the fiber while reducing the water solubility of the dye after coordination. The light fastness of the rhubarb-dyed silk fabrics was excellent, reaching grade 4. This is mainly because the chromophoric groups of the anthraquinone-based rhubarb dye are less prone to photo-oxidation under ultraviolet and visible light irradiation, exhibiting good photostability and high light fastness.

#### 2.4.2. UV-Protective Properties

Rhubarb dyeing significantly improved the UV-protective performance of silk. As shown in Table 3, undyed silk exhibited high UVA and UVB transmittance, with a UPF of only 3.44, indicating almost no UV-protection capability. After direct dyeing with rhubarb, UVA and UVB transmittance decreased notably, and the UPF increased to 18.72, corresponding to a “good” protection rating according to AS/NZS 4399:1996. All four metal post-mordants further reduced UV transmittance. In particular, Fe^2+^-mordanted samples gave UPF values above 25, rated as “very good,” with both UVA and UVB transmittance below 5%. This improvement is closely related to the molecular structure of rhubarb pigment, which is an anthraquinone derivative containing multiple carbonyl and hydroxyl groups [23,25]. This structure can absorb high-energy UV radiation, undergo photochemical reactions, and restructure, converting UV energy into other forms to achieve UV-blocking effects [25]. Mordanting promotes the binding of rhubarb pigment molecules to silk, increasing the amount of pigment on the fabric and thereby enhancing UV-protective performance.

#### 2.4.3. Antibacterial Properties

**Figure 7:**
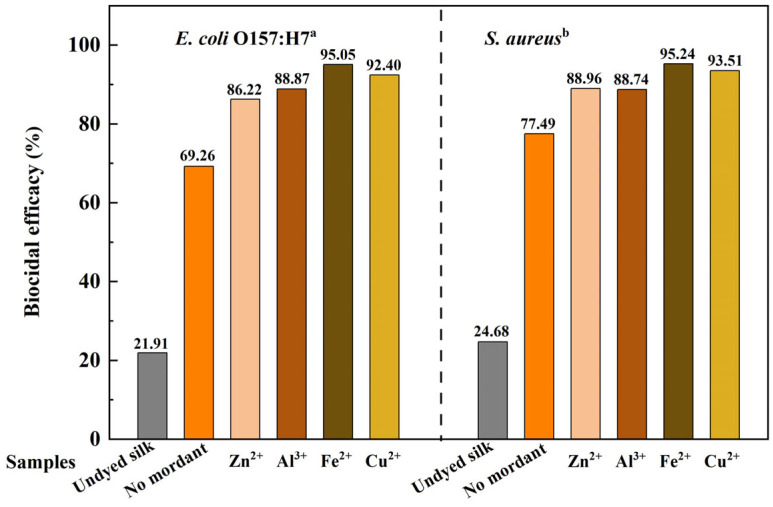
Antibacterial efficacies of the silk before and after dyeing with different mordants. a: The inoculum of *E. coli*; b: *S*. *aureus* were 2.83 × 10^7^ and 2.31 × 10^7^ CFU per swatch.

Rhubarb dyeing imparted good antibacterial activity to silk. As presented in Figure 7, the inhibition rates of undyed fabric against *E. coli* and *S. aureus* were 21.91% and 24.68%, respectively. After direct dyeing, these rates increased to 69.26% and 77.49%, indicating that rhubarb-dyed silk possesses considerable antibacterial efficacy. This is mainly because anthraquinone derivatives in rhubarb pigment can interfere with bacterial sugar metabolism and intermediate-product oxidation/dehydrogenation, inhibiting protein and nucleic acid synthesis [26].

Post-mordanting with the four metal ions further enhanced antibacterial performance to varying degrees. Fe^2+^ and Cu^2+^ mordants showed the most significant effects, raising the inhibition rates against *E. coli* and *S. aureus* above 90% (95.05% and 92.40% for Fe^2+^; 95.24% and 93.51% for Cu^2+^). This enhancement can be attributed to two synergistic factors. First, the metal ions themselves possess intrinsic antibacterial activity: Cu^2+^ and Zn^2+^ are well-documented for their ability to disrupt bacterial cell membranes, interfere with enzymatic functions, and generate reactive oxygen species; Fe^2+^ contributes through Fenton chemistry, producing toxic hydroxyl radicals; even Al^3+^, though less potent, can affect bacterial growth by interfering with membrane transport and enzyme activity at higher concentrations. Second, the formation of metal–dye complexes through coordination between the anthraquinone structure and metal ions likely alters the bioavailability and release kinetics of the active species, enhancing their interaction with bacterial targets. The particularly strong performance of Fe^2+^- and Cu^2+^-mordanted samples thus reflects the combination of potent intrinsic activity of these ions and the stable complexation with the dye, which ensures prolonged and effective antibacterial action.

## 3. Materials and Methods

### 3.1. Materials and Reagents

Rhubarb (*Rheum officinale* Baill.) was purchased from Shuo Zhen Tang Agricultural Products Co., Ltd. (Bozhou, China). Silk fabric (03 Crepe de Chine) was supplied by Suzhou Jiadoli Silk Garment Co., Ltd. (Suzhou, China). Sodium hydroxide, anhydrous aluminum sulfate, zinc sulfate heptahydrate, copper sulfate anhydrous, sulfuric acid, and ferrous sulfate heptahydrate were all of analytical grade. Sodium hydroxide was obtained from Xilong Scientific Co., Ltd. (Foshan, China); other reagents were purchased from Sinopharm Chemical Reagent Co., Ltd. (Shanghai, China) or Shanghai Aladdin Bio-Chem Technology Co., Ltd. (Shanghai, China) *Staphylococcus aureus* (ATCC 6538) and *Escherichia coli* (ATCC 8099) were provided by Vita Chemical Reagent Co., Ltd., Shanghai, China.

### 3.2. Extraction of Functional Pigment from Rhubarb

The pigment was extracted using a solvent-immersion method. Briefly, 10 g of dried rhubarb powder was placed in a glass bottle and extracted in a thermostatic water bath. Single-factor experiments were conducted to investigate the effects of pH (3–11), temperature (40–90 °C), time (10–60 min), and number of extraction stages (1–5) on extraction efficiency. After extraction, the mixture was cooled to room temperature, centrifuged twice, and vacuum-filtered to obtain a clear extract. The extract was diluted appropriately, and its absorbance at 400 nm was measured using a UV-8000S double-beam UV-Vis spectrophotometer (Shimadzu (China) Co., Ltd., Shanghai, China). (scan range 300–800 nm) to calculate the extraction yield. Multi-stage extraction was performed under optimal conditions until the extract color faded noticeably, thereby determining the ideal number of extraction stages.

### 3.3. Dyeing of Silk Fabric

#### 3.3.1. Pre-Treatment

The silk fabric (03 Crepe de Chine) was purchased as ready-for-dyeing (RFD) material, which had been pre-scoured and degummed by the supplier. Prior to dyeing, the fabric was conditioned at 20 ± 2 °C and 65 ± 2% relative humidity for 24 h to achieve moisture equilibrium. No further chemical pre-treatment was performed.

#### 3.3.2. Direct Dyeing

Dyeing was carried out in an HT-12p infrared high-temperature dyeing machine (Nantong Sansi Electromechanical Technology Co., Ltd., Qidong, China) with automatic reversal at 25 r/min. The dyeing started at 30 °C, then rose to 80 °C at 2 °C/min and held for a set time. The effects of dye-bath pH (3–11), liquor ratio (1:20–1:50), and dye-solution dilution ratio (0–8×) on dyeing performance were systematically examined. After dyeing, the fabric was rinsed with tap water, washed at 40 °C (liquor ratio 50:1), rinsed with cold water, and air-dried at room temperature.

#### 3.3.3. Post-Mordanting with Metal Ions

**Figure 8:**
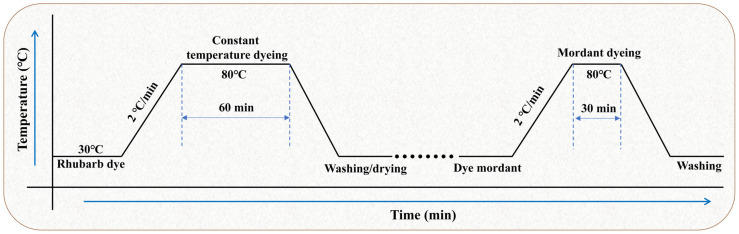
A process curve of step-by-step rhubarb dye dyeing and mordanting of silk fabric.

Based on the optimized direct dyeing conditions (liquor ratio 1:40, pH 7, 80 °C, 60 min), post-mordanting was performed using metal salts (ZnSO_4_, CuSO_4_, Al_2_(SO_4_)_3_, FeSO_4_) at concentrations of 3% (owf). The pre-dyed wet fabric was treated in the mordant bath (pH 7, liquor ratio 1:40) at 80 °C for 30 min, followed by rinsing, soaping, and air-drying. The overall process of dyeing experiment is shown in Figure 8.

### 3.4. Testing and Characterization

#### 3.4.1. Color Measurement

Color coordinates (L, a, b, C) and K/S values were measured using a Datacolor Spectro 1050 spectrophotometer (Guangzhou Chenglidong Instrument Co., Ltd., Guangzhou, China). Five readings were taken per sample and averaged.

#### 3.4.2. Durability Analysis of Dyed Fabrics

The color fastness to washing of the dyed silk fabrics was evaluated using a Wash TecP washing fastness tester (Ningbo Textile Instrument Factory, Ningbo, China) according to ISO 105-C06 [32]. The dyed silk fabric was stitched together with two standard lining fabrics, placed in a solution containing soap and anhydrous sodium carbonate, mechanically agitated under specified time and temperature conditions, then rinsed and dried. The color change of the dyed fabric and the staining of the lining fabrics were assessed using gray scale standards.

The color fastness to rubbing was determined following ISO 105-X12 [33] using a Model 670 rubbing fastness tester (Ningbo Textile Instrument Factory). A white rubbing cloth was rubbed back and forth against the dyed silk fabric under defined conditions. After a specified number of rubbing cycles, the degree of color transfer to the rubbing cloth was evaluated to determine the rubbing fastness.

The color fastness to light was assessed in accordance with ISO 105-B02 [34] using an Atlas Xeno Test Alpha light fastness tester (Ningbo Textile Instrument Factory). The silk fabric was exposed under specified conditions to an artificial light source simulating daylight (D65). The color change was then compared with blue wool references to evaluate the light fastness rating.

#### 3.4.3. UV-Protective Performance

The ultraviolet protection factor (UPF) and transmittance (T%) in the UVA (315–400 nm) and UVB (280–315 nm) ranges were determined with a UV-1000F textile ultraviolet tester (Shanghai Ultra Blue Scientific Inc Technology Co., Ltd., Shanghai, China). Five measurements were averaged per sample.

#### 3.4.4. Antibacterial Activity

Antibacterial performance was evaluated according to a modified AATCC 100-2004 standard, with undyed silk as the control [35,36]. Two swatches (2.56 cm × 2.56 cm) were used per test: 25 μL of bacterial suspension was placed on one swatch, covered with the second swatch, and pressed for full contact. After 30 min, the swatches were vortexed in PBS, serially diluted, plated on agar, and incubated at 37 °C for 24 h for colony counting. The bactericidal rate was calculated based on initial and final colony counts. Each test was repeated three times and the results were averaged.

## 4. Conclusions

This study systematically optimized the extraction of functional pigment from rhubarb and evaluated its application in dyeing silk fabrics. The optimal extraction conditions were: solid-to-liquid ratio 1:20, 80 °C, 50 min, pH 11, with three extraction stages, yielding high pigment efficiency. For direct dyeing, the best results were obtained at pH 7, bath ratio 1:40, 5% dye concentration, 80 °C for 60 min, leading to a high K/S value and uniform coloration. Post-mordanting effectively tuned color and depth: FeSO_4_ gave deep gray with the highest K/S increase, CuSO_4_ produced brown with notable K/S improvement, ZnSO_4_ shifted hues to yellow with minimal K/S change, and Al_2_(SO_4_)_3_ provided bright yellow though with slightly reduced K/S. The dyed fabrics exhibited greatly enhanced UV protection, with UPF increasing from 3.44 (undyed) to 18.72 (good) after direct dyeing and to 29.80 (very good) after Fe^2+^ mordanting, both showing <5% UVA/UVB transmittance. Additionally, rhubarb-dyed silk showed strong antibacterial activity, achieving inhibition rates of 69.26% (*E. coli*) and 77.49% (*S. aureus*) after direct dyeing, which exceeded 95% after Fe^2+^ or Cu^2+^ mordanting. The optimized extraction and dyeing conditions (moderate temperature, short time, reusable extraction stages) of this work contribute to process economy, and supports the use of rhubarb as a functional natural dye for producing multifunctional silk textiles suitable for high-end apparel and medical applications.

## Figures and Tables

**Table 1:** Effect of metal ion coordination on the K/S value and color characteristic value of rhubarb dye-dyed silk fabrics.

Dyeing Method	K/S Value	CIEL*a*b* and CIEL*c* Values			
		L	a*	b*	c*
No mordant	2.52	57.98	7.38	45.61	46.21
Zn^2+^ mordant	2.61	43.05	11.19	25.54	27.88
Al^3+^ mordant	1.61	56.71	6.5	35.6	36.19
Fe^2+^ mordant	5.16	21.96	0.63	6.09	6.12
Cu^2+^ mordant	4.6	31.21	10.24	19.44	21.97

**Table 2:** Color fastness of rhubarb extract-dyed silk fabrics before and after mordant dyeing.

Dyeing Method	Rubbing Fastness		Washing Fastness		Light Fastness
	Dry	Wet	Fade	Staining	
No mordant	3–4	3	2–3	3–4	4
Zn^2+^ mordant	4–5	4	4	4–5	4–5
Al^3+^ mordant	4–5	3–4	3–4	4–5	4–5
Fe^2+^ mordant	4	3	3	4	4
Cu^2+^ mordant	4	3–4	3–4	4	4–5

**Table 3:** UV-protection indices of silk before and after dyeing with different mordants.

Dyeing Method	T (UVA, %)	T (UVB, %)	UPF
Undyed silk	42.96	21.16	3.44
No mordant	6.91	5.06	18.72
Zn^2+^ (5%)	5.07	4.31	22.86
Al^3+^ (5%)	7.48	4.86	19.31
Fe^2+^ (5%)	3.44	3.35	29.80
Cu^2+^ (5%)	4.62	4.34	22.84

## Data Availability

Data are contained within the article.
